# Oligometastatic Pancreatic Cancer to the Liver in the Era of Neoadjuvant Chemotherapy: Which Role for Conversion Surgery? A Systematic Review and Meta-Analysis

**DOI:** 10.3390/cancers12113402

**Published:** 2020-11-17

**Authors:** Ottavia De Simoni, Marco Scarpa, Marco Tonello, Pierluigi Pilati, Francesca Tolin, Ylenia Spolverato, Mario Gruppo

**Affiliations:** 1 Unit of Surgical Oncology of the Esophagus and Digestive Tract, Veneto Institute of Oncology IOV-IRCCS, via Gattamelata, 64-35128 Padova, Italy; ottavia.desimoni@iov.veneto.it (O.D.S.); marco.tonello@iov.veneto.it (M.T.); pierluigi.pilati@iov.veneto.it (P.P.); francesca.tolin@iov.veneto.it (F.T.); 2 Department of Surgery, Oncology and Gastroenterology, University Hospital of Padua, via Giustiniani, 2-35128 Padova, Italy; marco.scarpa@aopd.veneto.it (M.S.); yleniacamilla.spolverato@aulss6.veneto.it (Y.S.)

**Keywords:** pancreatic cancer, oligometastatic disease, initial chemotherapy, conversion surgery, liver metastases

## Abstract

**Simple Summary:**

The development of new polychemotherapy regimens in patients with metastatic pancreatic cancer (mPDAC) have demonstrated significant improvement in clinical outcome, but evidence of the role of surgery following a favorable response to initial chemotherapy (IC) is still poor. The aim of the study is to analyze the impact of surgery following IC on survival in mPDAC, focusing on oligometastatic disease to the liver. Data retrieved from available literature confirm increased survival in selected oligometastatic patients treated with surgery + IC compared to IC alone (23–56 months vs. 11–16.4 months), suggesting a potential role for conversion surgery in a tailored and multimodality approach to pancreatic cancer patients. Better knowledge of tumor biology and a wide consensus on diagnostic criteria could lead to the consideration of oligometastatic disease as a particular and different stage of disease.

**Abstract:**

*Background*: the improved survival rates achieved using new polychemotherapy regimens in patients with metastatic pancreatic cancer (mPDAC) have suggested a potential role for surgery following a favorable response to initial chemotherapy (IC). The purpose of this systematic review is to summarize the available evidence on the role of surgery following IC in mPDAC, focusing on oligometastatic disease to the liver (lmPDAC). *Methods*: studies reporting on patients with lmPDAC undergoing surgery after IC were included. The main outcome was overall survival (OS). *Results:* six observational retrospective studies were included in the qualitative analysis. Data were retrieved on 2087 patients. The most common IC regimen in patients undergoing surgery was FOLFIRINOX (N 84, 73%). Only three studies reported survival comparison among patients treated with IC+surgery vs. IC alone. Median OS varied from 23 to 56 months after conversion surgery vs. 11 to 16.4 months after IC alone. *Conclusions:* despite wide heterogeneity of chemotherapy regimens, different downstaging criteria and potential selection biases, patients with oligometastatic lmPDAC undergoing surgery after IC have significantly higher survival rates compared to patients treated with IC alone. Future trials are needed for definition of univocal criteria of downstaging, oligometastatic definition and indications for surgery.

## 1. Introduction

Pancreatic cancer is currently the third leading cause of cancer mortality in the USA, and projections to 2030 estimate that the disease will become the second leading cancer-related cause of death, after lung cancer [1,2,3]. Complete surgical resection with adjuvant systemic chemotherapy currently provides the only chance of a curative treatment option and long-term survival for pancreatic cancer patients. Unfortunately, only 15–20% of patients are diagnosed early enough to be resectable [4,5], and about 50% of the patients are diagnosed at a metastatic stage [6]. Furthermore, most of the patients with localized disease at diagnosis develop metastases at a later time; pancreatic cancer commonly spreads to the liver, lungs, lymph nodes, peritoneum and adrenal gland [7,8,9]. Overall, up to 70% of patients show liver metastases from ductal adenocarcinoma of the pancreas (lmPDAC) along the course of their disease [10].

Nowadays, systemic chemotherapy plays the main role in patients with metastatic pancreatic cancer (mPDAC). 

In the past, Gemcitabine has been the reference treatment for advanced pancreatic cancer [11]. Subsequently, in patients with advanced pancreatic cancer, one year survival has slightly improved because of the wider use of systemic chemotherapy and, more recently, the use of polychemotherapy regimens; the combination of chemotherapy with folinic acid, 5-fluorouracil, irinotecan and oxaliplatin (FOLFIRINOX) demonstrated a significant improvement in overall survival (OS) in subjects with good performance status, compared to Gemcitabine alone [12]. Similarly, an improvement in OS was demonstrated in patients with mPDAC treated with the combination Gemcitabine/Nab-Paclitaxel compared with Gemcitabine alone [13]. Moreover, adjuvant chemotherapy with gemcitabine plus capecitabine increased overall survival compared with gemcitabine alone after resection for pancreatic cancer [14].

Although palliative chemotherapy is the standard of care for patients with mPDAC, a review by Ghidini et al. suggested a rationale for additional liver surgical or locoregional treatment in oligometastatic PDAC, based upon a comprehensive summary of recent literature [15]. 

The term “oligometastases” was first proposed in 1995 and was related to patients with limited metastatic tumors; this concept was developed, according with the natural history of cancer progression, in order to describe an intermediate stage of disease, between localized and systemic conditions, and potentially suitable for additional local or locoregional treatments with curative intent [16]. 

Finally, the progressive advances in surgical techniques and results have allowed more patients to undergo surgical resection [17]. High-volume medical centers are increasingly proposing chemotherapy prior to surgery in order to downsize locally advanced tumours to obtain resectable neoplasm; the improved response rate and survival achieved using polychemotherapy regimens in patients with mPDAC have promoted the new concept of neoadjuvant therapy followed by resection for patients with locally advanced pancreatic cancer [12,13]. 

New evidence has proposed FOLFOXIRI as a possible neoadjuvant therapy in selected advanced cases, in particular in oligometastatic disease [18,19,20,21]. In recent years, for patients with pancreatic cancer metastatic to the liver, there has been increased interest in exploiting strategies that have successfully been used for the treatment of oligometastases in other tumor types [15].

However, the management of the subgroup of patients with pancreatic cancer oligometastatic to the liver is still not clear and it should be considered a new challenge; furthermore, no randomized controlled trials are available, which have confirmed superiority of any of the applied therapies, and no evidence-based recommendation or international consensus is available on this topic yet.

The purpose of this systematic review is to summarize the available evidence and the role of initial chemotherapy in the treatment of mPDAC, focused on pancreatic cancer oligometastatic to the liver, in terms of multiple survival outcomes. 

## 2. Materials and Methods 

### 2.1. Study Design

We performed a systematic literature search, study design and data analysis following PRISMA (Preferred Reporting Items for Systematic Reviews and Meta-Analyses) guidelines (see Supplemental Contents—PRISMA checklist, http://links.lww.com/MD/ B316) [22]. The protocol of this meta-analysis was registered with the prospective register of systematic reviews, PROSPERO (CRD42020135654).

### 2.2. Search Strategy

Five medical databases were consulted in this research: Medline, Embase, the Cochrane Database of Systematic Reviews, Web of Science and Scopus. 

The primary search strategy included keywords and medical subject headings as follows: “Pancreatic cancer”, “Pancreatic cancers”, “Cancer of pancreas”, “Cancer of the pancreas”, “Duct cell carcinoma of the pancreas”, “Ductal carcinoma of the pancreas”, “Metastasis”, “Metastases”, “Neoplasm metastases”, “Neoplasm metastasis”, “Oligometastatic”, “Neoadjuvant therapies”, “Neoadjuvant therapy”, “Neoadjuvant treatment”, “Neoadjuvant treatments”, “Chemotherapies”, “Chemotherapy”, “Cancer chemotherapy protocol”, “Cancer chemotherapy protocols”, “Metastasectomy”, “Metastasectomies”, “Pancreaticoduodenectomy”, “Pancreaticoduodenectomies”, “Distal pancreatic resection”, “Pancreatic resection”, “Extended pancreatic resection”, “Lymphadenectomy”, “Lymphadenectomies”.

Articles from the search results have been selected independently by two authors (M.G and O.D.) following the inclusion and exclusion criteria. Any disagreements in study inclusion between the two authors was resolved by discussion. Only clinical studies written in English, Dutch, Spanish, German, French or Italian were selected. Studies with human patients 18 years of age and above were eligible for inclusion in the review. We did not include data quoted as unpublished or derived from abstracts. 

### 2.3. Selection Criteria and Outcome Measures

We included all studies investigating a series of patients with oligometastatic pancreatic cancer who underwent pancreatic resection, metastasectomy and/or RFA after neoadjuvant chemotherapy or preoperative chemotherapy. In the case of duplicate publications that reported on (parts of) similar patient data, only the most recent and complete data sets were considered. According to the PICOS criteria, articles were selected in this systematic review according to the follow eligibility criteria: 1. Participants: adults with metastatic pancreatic adenocarcinoma; 2. Intervention: pancreatic resection, metastasectomy and/or RFA after initial chemotherapy; 3. Comparison: patients with mPDAC undergoing only systemic chemotherapy; 4. Outcomes: the main outcome measure was overall survival. Secondary outcome measures were: postoperative mortality and morbidity. We then focused on the subgroup of patients with pancreatic cancer oligometastatic to the liver. Meta-analyzed endpoint was overall survival. All relevant text, tables and figures were reviewed for data extraction.

### 2.4. Quality Assessment of Retrieved Articles

Two researchers independently assessed the quality of the articles using a quality evaluation list constructed with predefined parameters including: number of patients, accurate description of surgical procedures, accurate description of chemotherapy treatment and whether the study series was consecutive. Moreover, the Newcastle−Ottawa Scale (NOS) was utilized for assessing the quality of non-randomized studies in meta-analyses. 

### 2.5. Data Extraction

Data were extracted from original articles only using a set of predetermined parameters: demographic data, localization of cancer, histological details of adenocarcinoma, type of surgery, type of neoadjuvant chemotherapy, morbidity, 90 days mortality, overall survival.

### 2.6. Statistical Analysis

Review Manager 5.3 (Cochrane Collaboration, Nordic Cochrane Centre, Copenhagen, Denmark) was used for data analysis. All statistical measures were assessed with *p* 0.05 significance level. The I^2^ statistic was used to determine the heterogeneity of the included studies. Low, moderate and high heterogeneity was considered for levels of I^2^ values of 25–49%, 50–74%, and above 75%, respectively [23]. We used mean difference analysis. The graphical description of the statistical results was illustrated with forest plot. Evaluation of publication bias was determined with funnel plot analyses. 

## 3. Results

### 3.1. Study Selection

**Figure 1:**
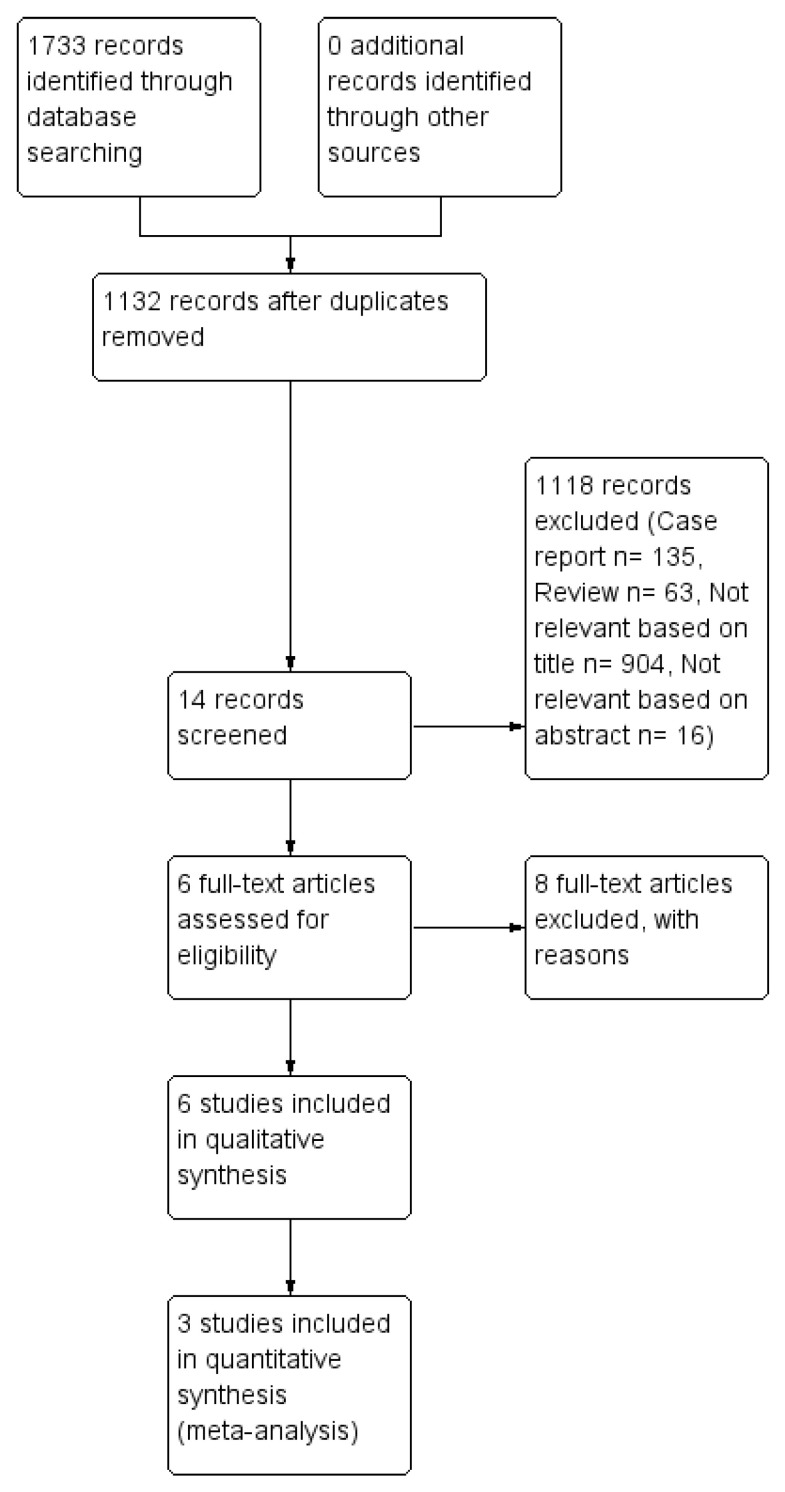
Flow chart of studies selection.

After the literature search, 1132 relevant non-duplicated records had been identified, 920 of them were excluded based on the title or the abstract because they covered a variety of irrelevant topics. A further 198 articles were then excluded because 135 were case reports and 63 were reviews. Finally 6 studies, published between 2016 and 2019, matched the selection criteria and have been included in the quality analysis, as shown in Figure 1 [24,25,26,27,28,29]. Authors of potentially eligible studies with minor missing or incomplete data, were directly contacted and invited for additional information and data. Studies from authors that have answered with updated and complete data, have been included in the analysis. Therefore, 3 studies were included in the quantitative synthesis (meta-analysis) [25,26,27].

### 3.2. Study Characteristics and Patients Characteristics 

Six studies, all published between 2016 and 2019, matched the inclusion criteria and have been included in the qualitative analysis [24,25,26,27,28,29]. All the included studies were observational retrospectives. The demographic characteristics of the included studies are shown in Table 1.

All studies had a quality score ≥ 6, assessed using Newcastle−Ottawa score. Moreover all the studies reported an accurate description of surgical procedure and chemotherapy treatment, except for two [27,29]. The level of evidence and study quality have been summarized in Table 2.

Patient characteristics are reported in Table 3. A total number of 2087 of patients with mPDAC were analyzed. Some 115 patients with mPDAC underwent surgery after IC. The most common chemotherapy regimen utilized in patients with mPDAC undergoing surgery was FOLFIRINOX, for 84 (73%). In two studies, FOLFIRINOX was the only chemotherapy regimen used [26,29]. The interval between last chemotherapy and surgery varied from a median of 2 to 12 months. In all studies the main metastatic localization in patients undergoing surgery after IC was the liver for 85 (79%) patients. Others metastatic localizations were: lung (N 7, 6%), extraregional lymph nodes (N 3, 2%), peritoneum (N 9, 10%). Based on available data both pancreaticoduodenectomy and distal pancreatectomy and total pancreatectomy were well represented. Surgery on liver metastases was heterogeneous, with a predominance of atypical resections.

As shown in Table 4, only 5 studies presented differentiated survival data for patients with pancreatic cancer metastatic to the liver undergoing IC [24,25,26,27,29]. The median age of all patients with lmPDAC ranged from 57.5 to 65 years. The median value of CA19.9 at diagnosis ranged from 210 to 1575 U/mL in patients who had undergone IC and surgery vs. 525–1846 U/mL in subjects treated with chemotherapy only. Other demographics and pathological parameters (gender, ECOG, tumor size) were not retrievable. Most authors described the rigorous criteria of response to initial chemotherapy used to select patients for surgery after IC. Only one article had, as a criteria of response to initial chemotherapy, a complete response of liver metastasis after initial chemotherapy [24]. The percentage decrease of CA19.9 in patients with lmPDAC undergoing surgery after IC varied from 92% to 97%. The percentage decrease of CA19.9 in patients with lmPDAC undergoing only IC ranged from 42% to 60%.

The most used chemotherapy regimen in patients who had undergone surgery after IC was FOLFIRINOX (N 50, 75%). Limited to the available data, it was not possible to stratify survival outcomes based on different chemotherapy regimens. The number of total patients selected for surgery after IC was N 66 (8%). The proportion of patients selected for surgery after IC varied from 4.5% to 52.6%. Data on morbidity and mortality are shown in Table 4. Only two articles described complications after surgery [24,25]. The median OS in patients with lmPDAC undergoing surgery after IC varied from 23.25 to 56 months. However, the median OS in patients with lmPDAC undergoing only IC varied from 11 to 16.4 months. 

### 3.3. Meta-Analysis: Survival Analysis

**Figure 2:**
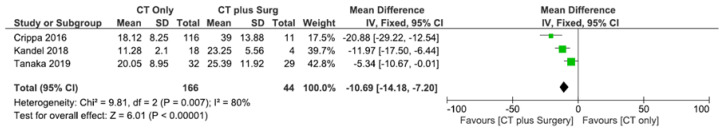
Forest plot of survival analysis.

Three studies described comparable patient groups in terms of outcome of survival and thus, a metanalysis was attempted [25,26,27]. All three studies investigated patients with pancreatic cancer metastatic to the liver undergoing IC followed by surgery or not and assessed an intervention group versus a control group. In these studies, cumulatively, 210 patients have been evaluated, of which 44 underwent surgery after IC and 166 underwent only chemotherapy. Heterogeneity resulted as significantly high among these studies (I2: 80%, *p* < 0.007) so the different dataset could not be pooled. As graphically shown in Figure 2, OS was longer in patients with pancreatic cancer with liver metastasis who underwent IC followed by surgery, compared to subjects treated only with chemotherapy (MD −10.69, 95% CI −14.18–−7.2, *p* < 0.00001). 

### 3.4. Sensitivity Analysis and Publication Bias 

**Figure 3:**
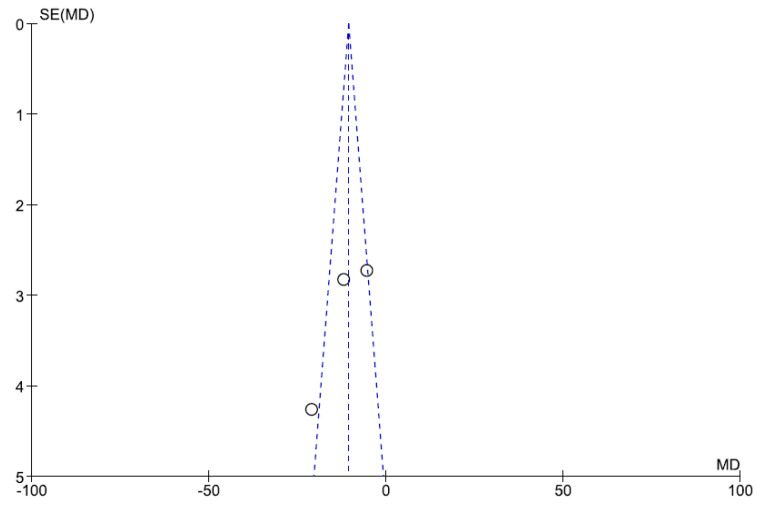
Funnel plot.

Heterogeneity was high among studies (I^2^ = 80%, *p* = 0.007). Publication bias was evaluated with funnel plot analyses, as shown in Figure 3. 

## 4. Discussion

The insidious onset and aggressiveness of pancreatic cancer mean that only 15–20% of patients have the chance to receive curative surgery at first diagnosis [1,4]. Moreover, about 50% of patients with pancreatic cancer are diagnosed at metastatic stage [6]. Among these, the 5-year survival rate is only 2%, while reported 1-year survival rates with Gemcitabine varied from 17 to 23% [3,11,12,30].

Despite advances in surgical techniques and oncology over the last decades, pancreatic cancer and its metastatic condition continues to pose major therapeutic challenges.

In recent years, the potential role of surgery in mPDAC has been explored. If some authors have stated that surgery is not beneficial in this setting, others, such as Shrinkade et al., provided initial and encouraging results, reporting a median survival of 13.8 months in patients with metastatic pancreatic cancer who had undergone pancreatic resection [31].

Gemcitabine alone was the cornerstone of medical treatment for nearly 15 years and demonstrated a positive impact on survival [11]. Afterwards, the introduction of polychemotherapy regimens has brought further benefits.

In recent years, a randomized phase III trial, MPACT, showed that a combination of gemcitabine and nab-paclitaxel (Gem + nabPTX) yielded a statistically significant survival benefit and response rate when compared with gemcitabine monotherapy [13].

Moreover, as underlined by PRODIGE/ACCORD 11, the FOLFIRINOX regimen has been evaluated in metastatic pancreatic adenocarcinoma and was an effective first-line treatment option, associated with an increase in overall survival, disease-free survival and response rate in comparison to gemcitabine alone [12,13]. Many centers assessed the efficacy of FOLFIRINOX and other polychemotherapy regimens as neoadjuvant treatment in locally advanced disease (LAPC) and, recently, in borderline resectable pancreatic cancer (BRPC). Surely, one of the main purposes of neoadjuvant therapy for BRPC is to achieve margin-negative resection and eliminate micrometastatic cells prior to surgery, preventing metastatic recurrence [32]. Miyasaka suggested that neoadjuvant with Gemcitabine plus nab-paclitaxel in BRPC potentially decreased recurrence during the intermediate-term postoperative period (33% vs. 77%) with a significantly higher R0 resection rate (100% vs. 77%, *p* = 0.0100) [33]. Moreover, Ferrone underlined that FOLFIRINOX as neoadjuvant chemotherapy in LAPC or BRPC had a significant decrease in lymph node positivity (35% vs. 79%), perineural invasion (72% vs. 95%) and a significant increase in overall survival compared with resectable patients who underwent exploration without neoadjuvant treatment (*p* = 0.008) [34]. Furthermore, a recent randomized phase III trial ESPAC 4, showed that the adjuvant combination of gemcitabine and capecitabine could be the new standard of care following resection for pancreatic ductal adenocarcinoma [14]. 

At the same time, surgical techniques have also substantially improved, allowing more patients to undergo resection. 

An emerging multimodality approach with a combination of surgery and greater use of polychemotherapy regimens has brought up the potential role of conversion surgery in pancreatic cancer. In fact, not only do 50% of the patients at the first diagnosis have metastatic disease but also a significant proportion of patients are unable to initiate adjuvant chemotherapy following pancreatectomy, frequently because of postoperative complications or rapid disease recurrence; this evidence has suggested the opportunity to administrate chemotherapy before surgery with the aim to increase the number of subjects treated with CT and better select patients who can benefit from a multimodal approach, with the longer survival and better postoperative and pathological outcomes reported for those patients who underwent curative intent surgery after neoadjuvant treatment [35,36,37,38]. 

Beside a significant improvement in chemotherapy regimens and surgical techniques, the “oligometastatic disease” has become increasingly important for researchers. In 1995, Hellman and Weichselbaum first proposed the clinically relevant condition of oligometastasis, developing the idea of an intermediate stage of disease, between localized and spread systemic conditions [36]. The multistep progression of cancer and a better knowledge of tumoral behavior have supported this concept [37,38,39,40].

The purpose of this systematic review was to analyze the role of conversion surgery after initial chemotherapy in the treatment of a particular condition of metastatic pancreatic cancer: the oligometastatic disease to the liver.

Conversion surgery is currently adopted in other oligometastatic neoplasms such as colorectal cancer, with the aim to cure or improve control of the disease in selected patients with major response to initial chemotherapy or favorable molecular profiles [41,42,43]; furthermore, other aggressive advanced neoplasms, previously considered not suitable for surgery, such as gastric cancer, are demonstrating good outcomes after conversion surgery following initial chemotherapy in selected cases [44]. This evidence lets us speculate that a better knowledge of tumor biology, with detection of prognostic molecular biomarkers, and a careful evaluation of response to chemotherapy could lead to an increase in conversion surgery rates and a better selection of subjects who can benefit from it. 

The proper selection of surgical candidate patients after initial chemotherapy for lmPDAC is considered by most of the authors within this review the first and most important step. The decision for a surgical resection in patients with mPDAC to the liver has been made on a highly individual basis, including the patient’s wishes, age, clinical status, local resectability and the individual risk of complications [45]. In addition, the evaluation of the initial Eastern Cooperative Oncology Group performance status (ECOG) and its re-evaluation during a neoadjuvant treatment is fundamental for prognostic purposes. Furthermore, articles that emphasize the importance of initial chemotherapy also highlight the importance of finding the most appropriate criteria of response to initial chemotherapy (Table 4). 

Most of the authors share the fact that the downsizing criteria must include radiological criteria (response of the primary tumor and metastases) and biochemical criteria (decrease of the CA19.9 values). As suggested by Hong et al., the criteria of downsizing should be expanded in the future for the duration of the response and potential additional information with mutational analyses [19]. 

All the authors agree to define criteria for downsizing the achievement for resectability of the primitive neoplasm. However, the criteria for downsizing regarding the response of liver metastases are extremely heterogeneous: as reported in Table 4, Frigerio at al. considered surgical resection after restaging only in patients with radiological disappearance of liver metastasis, Crippa et al. performed resections in the case of a complete or major radiological response, while Tanaka accepted a maximum limit of six liver metastases [24,25,26]. 

From the data reported, only a small percentage of patients undergoing initial chemotherapy were finally selected for surgical treatment. From the available data only 66 patients out of 799 (8.2%) undergoing initial chemotherapy met the criteria of downstaging for surgery.

Frigerio et al. described how, by adhering to strict downstaging criteria, few patients (N 24, 4.5%) could be selected for surgery on the primary neoplasm, reporting a median overall survival of 56 months (36–75) [24]. Crippa et al. considered eligible for surgery patients who had a good response to initial chemotherapy, with a maximum of one liver metastasis (N 11, 8,5%), reporting a median overall survival of 39 months [25]. Tanaka et al. considered eligible for surgery patients with a maximum of six metastatic lesions, reporting a median overall survival of 25.39 months (Table 4) [26].

In meta-analysis, unfortunately, heterogeneity was significantly high among the studies (I2: 76%, *p* < 0.02) so the different datasets could not be pooled. However, as graphically shown in Figure 3, patients with lmPDAC who underwent chemotherapy and surgery were able to reach prolonged OS, compared to the solely chemotherapy group; nevertheless, no significant conclusion can be drawn from such data, because patients treated only with chemotherapy were not eligible for surgery, due to insufficient response to medical treatment. Furthermore, selected studies were retrospective case series and no perspective controlled trials are currently available. 

Furthermore, considering the chemotherapy response, the heterogeneity of radiological downsizing the criteria of liver metastases goes hand in hand with the wide variability of definitions of oligometastatic disease among the authors, as underlined in Table 4. The condition of oligometastatic disease is considered the starting point in the selection of patients, but actually there does not exist a unique definition, as demonstrated in reported articles where definitions are heterogeneous and mainly based on quantitative, dimensional and resectability criteria (Table 3).

Finally, as underlined by Reyes, the definition of oligometastasis has been gradually evolved and, in the absence of data to guide decisions, treatment of oligometastatic disease is still considered as a quality-of-life oriented approach, choosing personalized treatments, with a reasonable risk to benefit ratio and taking into account the patient’s own will in guiding them toward conservative or aggressive therapy [46,47]. 

Despite of heterogeneity of definitions, the oligometastatic disease in pancreatic cancer has undoubtedly become a source of debate regarding its potential treatment, claiming a wide consensus about its definition and classification in order to select patients with metastatic pancreatic cancer who can take advantage of a curative treatment.

As far as we know this is the first review focused on surgical treatment of pancreatic cancer oligometastatic to the liver after initial chemotherapy.

However, some limitations should be considered when interpreting our data: firstly, included studies are retrospective and with mono-institutional cohorts; secondly, as stated before, assessment of a definition of oligometastatic disease is still heterogenous and the definition of standard criteria of downsizing for surgery is lacking.

The adoption of universal criteria for definition of oligometastatic disease and for chemotherapy response assessment are needed to propose well-designed prospective studies, in order to evaluate the best therapeutic paths in this kind of patients.

## 5. Conclusions

Despite the wide heterogeneity of chemotherapy regimens, different intervals between chemotherapy and conversion surgery and different downsizing criteria, selected patients with lmPDAC who present good PS, a major response to initial chemotherapy and a favorable tumor biology could undergo conversion surgery after initial chemotherapy and reach significantly better survival rates than patients treated with chemotherapy alone. 

This review confirms that oligometastatic pancreatic cancer can be considered a particular entity and a specific stage of disease, which deserves a specific evaluation, as previously reported [36,46]. 

Larger multicentric perspective studies are needed to define and validate a widely accepted definition of oligometastatic disease and to confirm the potential benefit of conversion surgery after initial chemotherapy at this stage of disease.

## Figures and Tables

**Table 1:** Characteristics of the studies.

References	Publication Year	Country	Inclusion Period	Type of Study	Study Design	No. of Patients Analyzed
Frigerio [24]	2017	Italy	2007–2015	Retrospective	Case series, two centers	535
Crippa [25]	2016	Italy	2003–2013	Retrospective	Case series, two centers	127
Tanaka [26]	2019	Germany	2001–2017	Retrospective	Case series, one center	101
Kandel [27]	2018	USA	2005–2015	Retrospective	Case series, one center	42
Wright [28]	2016	USA	2008–2013	Retrospective	Case series, two centers	1147
Byun [29]	2019	Japan	2011–2017	Retrospective	Case series, one center	337

**Table 2:** Level of evidence and quality assessment of the selected studies.

Authors, Publication Year	Total Patients	Accurate Description of Surgical Procedure	Accurate Description of Chemotherapy Treatment	Consecutive	Newcastle−Ottawa Score			
					Selection Maximum ****	Comparability Maximum *	Outcome Maximum ***	Score (Out of 8)
Frigerio [24], 2017	535	Yes	Yes	Yes	***	-	***	6
Crippa [25], 2016	127	Yes	Yes	Yes	***	-	***	6
Tanaka [26], 2019	101	Yes	Yes	Yes	****	*	***	8
Kandel [27], 2018	42	Yes	No	Yes	****	*	***	8
Wright [28], 2016	1147	Yes	Yes	Yes	****	*	***	8
Byun [29], 2019	337	No	Yes	Yes	****	*	***	8

Newcastle−Ottawa Quality Assessment Scale for Cohort Study. (*: the study met the criteria for a domain of the Newcastle-Ottawa Scale; -: the criteria were not met).

**Table 3:** Characteristics of patients with mPDAC undergoing surgery after initial chemotherapy.

References	Patients, N	Definition of Oligometastatic Disease or Inclusion Criteria for Surgery after IC	Patients Undergoing Surgery after IC, N (%)	Type of IC in Patients Undergoing Surgery, N (%)	Median Interval last IC-Surgery, Months (range)	Main Metastatic Localisation				Type of Surgery for Primary Lesion, N	Type of Surgery for Liver Metastases N	Adjuvant CT N (%)	30 Day Mortality N (%)	OS Months (Range)
						Liver, N	Lung, N	Lymphnodes, N	Peritoneum, N					
Frigerio [24]	535	Disappearance of liver metastasis on radiological examination	24 (4)	FOLFIRINOX N 16 (66), GEM N 5 (21), Gemcitabine + Nab-Paclitaxel N 3 (13)	2 (2–16) ***	24	0	0	0	PD N 14 DP N 10	None	N 15 (63)	Not specified	56 (36–75) **
Crippa [25]	127	Single metastasis remaining after initial chemotherapy	11 (8)	FOLFIRINOX N3 (27), GEMOX N2 (2), PDXG N1 (9), PEFG N1 (9), PEXG N4 (36)	12 (6–20) ***	11	0	0	0	PD N 6 DP N 5	Atypical resection N 1, Segmentectomy N 2	Not specified	Not specified	39 *
Tanaka [26]	101	Maximum of six metastatic lesions.	43 (42)	FOLFIRINOX N43 (100)	Not specified	30	1	3	7	PD N 16 DP N 19 TP N 8	Not specified	N 4 (9), Unknown 9 (21)	1 (2)	21.9 (12.7–20.5) ***
Kandel [27]	42	≤2 metastatic tumors total in liver or lung, each <4 cm	6 (14)	Not specified	Not specified	4	0	0	2	PD N 4 DP N 2	Hepatic resection N1, Hepatic resection and RFA n 2, Radioembolization N 1	6 (100)	0	32/4 (1.4–44.28) **
Wright [28]	1147	Not specified	23 (2)	FOLFIRINOX N14 (60.9), Gemcitabine based regimens N 9 (39.1)	9.7 (5.8–12.8) ***	16	6	0	2	PD 15 DP 8	Metastasectomy N 9	Not specified	0	18.2 (11.8–35.5) ***
Byun [29]	135	Single metastatic lesion or resectable lesion	8 (5)	FOLFIRINOX 8 (100)	Not specified	NS	NS	NS	NS	Not specified	Not specified	Not specified	Not specified	32 *

Values are * mean, ** mean (s.d.), *** median (range). IC: initial chemotherapy; CT: chemotherapy; PD: pancreaticoduodenectomy; DP: distal pancreasectomy; TP: total pancreatectomy; GEM: gemcitabine; GEMOX: gemcitabine and oxaliplatin; FOLFIRINOX: oxaliplatin, irinotecan, fluorouracil and leucovorin; PEXG/PDXG: cisplatin, capecitabine, gemcitabine plus either epirubicin (PEXG) or docetaxel (PDXG); PEFG: cisplatin, epirubicin, fluorouracil and gemcitabine. NS: not specified.

**Table 4:** Criteria of response to initial chemotherapy, inclusion criteria for surgery, IC regimens and outcomes of five series of patients with lmPDAC who underwent surgery after IC.

Criteria of Response to IC											
References	Patients	Primary Tumor	Liver Metastases	Level of CA19.9	Total Patients with lmPDAC, N	Patients Undergoing Surgery after IC, N (%)	Type of Initial Chemotherapy, N (%)	Complications after Surgery N (%)	Median Postoperative Stay Days (Range)	OS in Patients who Underwent IC+Surgery Months (Range)	OS in Patients who Underwent IC alone Months (Range)
Frigerio [24] (2017)	lmPDAC who responded to systemic chemotherapy and underwent successful surgery.	Not specified	Disappearance of liver metastasis on radiological examination	Normalization or significant reduction of CA19.9	535	24 (4.5)	FOLFIRINOX N 16 (66), GEM N 5 (21), Gemcitabine+Paclitaxel N 3 (13)	PF B N4 (16.5) PF C N1 (4) HEMORRHAGE N1 (4) SEPSIS N3 (12.5)	13 (7–40)	56 (36–75)°°	-
Crippa [25] (2016)	lmPDAC who responded to systemic chemotherapy and underwent successful surgery.	Resectable or borderline	Complete or a major radiological response of the liver metastases with a single metastasis remaining	Major biochemical response	127	11 (8.5)	FOLFIRINOX N3 (27), PDXG N1 (9), PEFG N1 (9), PEXG N4 (36), GEMOX N2 (2)	PF A N2	8	39°°	11°°
Kandel [27] (2018)	lmPDAC who underwent systemic chemotherapy, primary tumor resection, and metastasectomy and/or RFA	Not specified	Not specified	Not specified	Not specified	4 (28)	Not specified	Not specified	Not specified	23.25°°	11.76°°
Tanaka [26] (2019)	lmPDAC undergoing pancreasectomy and metastasectomy after FOLFIRINOX	No tumor progression, technically resectable disease (resectable or borderline resectable, as defined by the NCCN guidelines)	A maximum of six metastatic lesions	Not specified	57	30 (52.6)	All patients FOLFIRINOX	Not specified	13 (5–56)	25.39°°	16.4°°
Byun [29] (2019)	lmPDAC who responded to systemic chemotherapy and underwent successful surgery.	Response to initial chemotherapy per RECIST criteria (Version 1.1), absence of local tumor extension to the major vessel	Single metastatic lesion or considered as resectable	Tumor markers normalized (or metabolic uptake decreased in PET)	80	1 (1.25)	All patients FOLFIRINOX	None	Not specified	23°°	13°°

PF: pancreatic fistula (defined according to ISGPF criteria: grade A-B-C); lmPDAC: liver metastatic pancreatic adenocarcinoma; IC: initial chemotherapy; GEM: gemcitabine; GEMOX: gemcitabine and oxaliplatin; FOLFIRINOX: oxaliplatin, irinotecan, fluorouracil and leucovorin; PEXG/PDXG: cisplatin, capecitabine, gemcitabine plus either epirubicin (PEXG) or docetaxel (PDXG); PEFG: cisplatin, epirubicin, fluorouracil and gemcitabine. Values are overall survival from diagnosis, °° overall survival from surgery.
